# Biodiversity and Interannual Variation of Harmful Algal Bloom Species in the Coastal Sea of Qinhuangdao, China

**DOI:** 10.3390/life13010192

**Published:** 2023-01-09

**Authors:** Yang Chen, Lu Wang, Zhiliang Liu, Du Su, Yibo Wang, Yanping Qi

**Affiliations:** 1 Research Center for Marine Science, Hebei Normal University of Science and Technology, Qinhuangdao 066004, China; 2 Hebei Key Laboratory of Ocean Dynamics, Resources and Environments, Qinhuangdao 066004, China; 3 Laoshan Laboratory, Qingdao 266237, China; 4 North China Sea Environmental Monitoring Center, State Oceanic Administration, Qingdao 266033, China

**Keywords:** harmful algal bloom species, Qinhuangdao coastal sea, interannual variation, biodiversity

## Abstract

For the frequent occurrence of harmful algal blooms (HABs) in the Qinhuangdao coastal sea (QCS) of the Bohai Sea in summer, we tested the hypothesis that high-biodiversity HAB species exist in the area, and a series of censuses of HAB species were conducted in the QCS in the summers of 2014–2019. Through morphological identification, we found 100 algae species representing 42 genera in 3 phytoplankton phyla in this study, among which Bacillariophyta was the most dominant phylum. We also found that the population density of Dinoflagellata increased from 2016 to 2019. In total, 59 HAB species were annotated in this study, including 39 of Bacillariophyta, 18 of Dinoflagellata and 2 of Ochrophyta, of which 13 HAB species were reported in the Bohai Sea for the first time, and most HAB species were widely distributed in the QCS in summer. Notably, four dominant HAB species displayed unique temporal and spatial distribution characteristics, while their distribution ranges and population densities increased from 2014 to 2019. The distributions of five environmental factors were different in the QCS, while the temperature, salinity, and dissolved inorganic nitrogen might be the key environmental factors influencing the distribution of dominant HAB species in the summer. In conclusion, this study provides a detailed evaluation of phytoplankton diversity and interannual variation in the QCS. The existence of a high level of biodiversity of algal bloom species suggests the need for long-term monitoring in order to further study and prevent potential HABs.

## 1. Introduction

Harmful algal blooms (HABs) are caused by rapid growth and aggregation of algae in the ocean and in fresh water [1,2]. They can be further subdivided into different types, including the “red tide” and “brown tide” formed by microalgae and the “green tide” and “golden tide” formed by macroalgae [2,3,4]. Moreover, HABs can also be classified as toxic or non-toxic depending on the toxicity of the HAB species [4,5]. Over the past 50 years, HABs in offshore waters have been a marine ecological issue worldwide [4], causing serious damage to ecological structure and function, threating food safety and human health, and even influencing social and economic development [6,7].

The Bohai Sea is a semi-enclosed ocean area with an average depth of 18 m, and consists of Liaodong Bay, Bohai Bay, Laizhou Bay, the central Bohai Sea, and Bohai Strait. The special geographical location leads to the high environmental heterogeneity and phytoplankton biodiversity of Bohai Sea. The most common algae in the Bohai Sea are diatom species, followed by dinoflagellates; additionally, the red tide species also possesses high biodiversity [8]. Bohai Sea is historically well-known for frequent HABs, as its eco-environment could be heavily influenced by human activities, terrestrial runoff, and global climate change [8,9]. The earliest record of HAB in the Bohai sea could date back to the *Noctiluca scintillans* red tide in 1952 [10], and the phytoplankton species in the Bohai Sea were systematically analyzed for the first time through the National Marine Survey from 1958 to 1959 [8]. The frequency and scale of red tides in Bohai Sea have increased since the 1990s [9], and Liaodong Bay has suffered the most frequent red tides [11]. In the last two decades, the Bohai Sea has been struck by HABs at least 151 times, with a cumulative area more than 36,209 km^2^, which mainly occurred from May to November [8]. A total of 140 red tide species (belonging to 5 phyla) were found in the investigations carried out in the Bohai Sea from 1957 to 2019, including 78 species of diatoms and 52 species of dinoflagellates [8]. Our previous study identified 74 potential HAB species in the Bohai Sea during an expedition in the summer of 2019 [12]; however, this number may be underestimated due to the limitation of existing molecular marker databases. In 2021, at least 12 red tides occurred in the Bohai Sea, with an area of more than 6882 km^2^ and with the longest duration being 51 days. These were primarily caused by the toxic dinoflagellate species *Margalefidinium polykrikoides* and the non-toxic dinoflagellate species *Scrippsiella acuminate* (https://www.mnr.gov.cn/ (accessed on 18 July 2022)).

**Figure 1:**
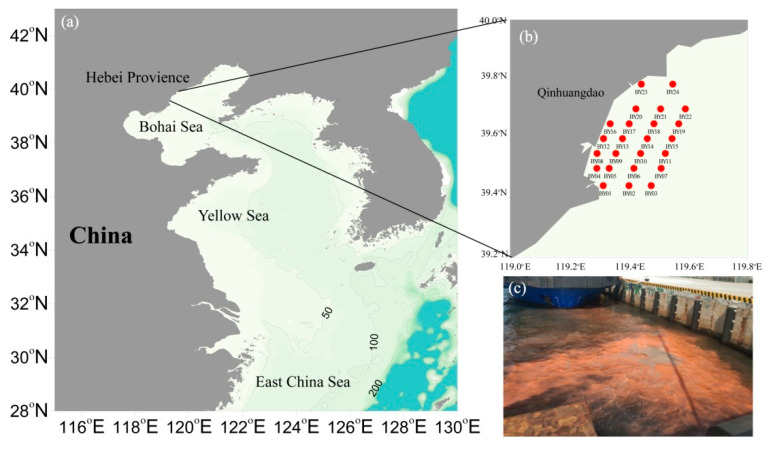
The location of sampling stations in the Qinhuangdao coastal sea. (**a**) Location of sampling sites in the Bohai Sea; (**b**) sampling sites in the summers of 2014–2019; (**c**) red tide of *Noctiluca scintillans* in the Qinhuangdao coastal sea in the summer of 2022.

As part of the Bohai Sea, Qinhuangdao coastal sea (QCS) has been struck by frequent HABs in recent years. From 2009 to 2017, 39 HAB events were reported in QCS alone, accounting for 45.9% of the HABs in the Bohai sea [13]. There, *Aureococcus anophagefferens* induced the brown tides in QCS from 2009 to 2015, resulting in a large number of scallop deaths in the breeding area, serious damage to local marine ecological environment, and significant economic losses in local aquaculture and the tourism industry [14,15,16]. In 2010, especially, the *Aureococcus anophagefferens* brown tide affected a large sea area of up to 3350 km^2^, leading to an economic loss of RMB 0.2 billion [17]. Amplification and sequencing analysis of 18S rDNA V9 regions of sediment samples showed that *Aureococcus anophagefferens* may be widely distributed in the world, and may have been present in the Bohai Sea for more than 1500 years [18]. It should be noted that large-scale *Aureococcus anophagefferens* blooms never occurred in China’s costal sea before 2008. Furthermore, after the frequent occurrences in 2009–2015, they have not been seen in the QCS since 2016. However, whether *Aureococcus anophagefferens* blooms will return to the Bohai Sea remains unknown. In contrast, the *Noctiluca scintillans* red tide in QSC was still observed in 2022 (Figure 1c), and has been intermittently going on for nearly 70 years in the Bohai Sea.

Heavily impacted by an intense coastal project, complex international shipping routes, and land-based pollution, QCS suffered greatly from a wide range of HABs with high frequency and persistence due to serious eutrophication of the seawaters [13]. It is well known that environmental factors could strongly affect the formation, expansion, and elimination of HABs, as well as the succession of the dominant HAB species. For instance, the dominant HAB species of the Yangtze Estuary in the spring has changed from diatoms to dinoflagellates over the past few decades, and phosphate (PO_4_^3−^) was considered the main factor affecting the succession of diatom blooms to dinoflagellate blooms [19]. Intensive human disturbance (e.g., industry, tourism, aquaculture, and sea reclamation) could change the environmental factors in QCS, thereby influencing the community structure and population density of phytoplankton in QCS, which is of great concern. Frequent outbreaks of multiple HABs (e.g., *Noctiluca scintillans* red tides, *Ulva prolifera* green tides [20], *Aureococcus anophagefferens* brown tides [18]) have resulted in huge economic losses in fisheries and the tourism industry in QCS. However, our understanding of the biodiversity, population density, and interannual variation of these HAB species is still limited. Unveiling the biodiversity and interannual variation of HAB species is essential for preventing ecological disasters in QCS.

In this study, a series of summer voyages were carried out from 2014 to 2019 to collect the phytoplankton samples in QCS. Microscopy was employed to determine the composition and abundance of algal species in QCS. Thereafter, the composition, biodiversity, geographical distribution, interannual variation, and impacting factors of HAB species were analyzed to deepen the understanding of the HAB outbreak mechanism in QCS.

## 2. Materials and Methods

### 2.1. Sampling Sites and Sample Collection in the Qinhuangdao Coastal Sea Area

During the summers (2–29 August) of 2014–2019, 122 seawater samples were collected at 24 sampling sites belonging to the 6 voyages (2014–2019) in the QCS (26.08–32.35° N, 120.94–125.90° E) (Figure 1). Sampling of this study was carried out onboard “Ji Yun 6666”, and detailed information on the six voyages is listed in Table S1.

In each sampling site, a phytoplankton net (76 μm) was used to trawl the seawater vertically from bottom to surface to collect the phytoplankton cells, and these were subsequently transferred to a 1 L specimen bottle with a final concentration of 2% formaldehyde. The algae species were identified and counted by an Olympus CX31 microscope (Olympus, Tokyo, Japan) using the Utermöhl method [21].

### 2.2. Collection and Determination of Environmental Factors

The temperature and salinity of seawater were measured in situ using a CTD system (SeaBird, Loveland, CO, USA). At each sampling site, water samples were collected using an SBE 32 Water Sampler (SeaBird, Loveland, CO, USA) at the surface and the bottom of the sea. At each depth, 500 mL seawater was collected and pre-filtered through a 200 μm mesh sieve to remove large plankton and abiotic substances. This was followed by filtering through 0.2 μm polycarbonate membranes (Millipore, Burlington, MA, USA) by a vacuum pump (Jinteng, Tianjin, China), and then the filtered seawater samples were transferred into sterile reagent bottles and stored at −20 °C before determination of the environmental factors. Meanwhile, a 0.45 μm membrane (GF/F, Whatman, Maidstone, UK) filtering a 500 mL seawater sample was transferred into a sterile tube and then stored at −20 °C for chlorophyll-a determination. A fluorometer (10-AU, Turner Designs, San Jose, CA, USA) and a continuous flow analyzer (QuAAtro39, SEAL Analytical, Norderstedt, Germany) were employed to determine the concentrations of chlorophyll-a and dissolved inorganic nutrients (i.e., PO_4_^3−^, NH_4_^+^, NO_3_^−^, and NO_2_^−^), respectively. The PO_4_^3−^ concentration and the total concentration of ammonia (NH_4_^+^), nitrite (NO_2_^−^), and nitrate (NO_3_^−^) were utilized to represent the concentrations of dissolved inorganic phosphorus (DIP) and dissolved inorganic nitrogen (DIN), respectively. The environmental factors (temperatures, salinities, chlorophyll-a, DIN, and DIP) of the sampling seawaters are listed in Table S1. The geographical profiles of the environmental factors are shown in Figure S1.

### 2.3. Taxonomic Annotation and Bioinformatics Analysis

The identification and annotation of each algal species in this paper was confirmed on the website AlgaeBase (http://www.algaebase.org (accessed on 13 August 2022)). Each HAB species annotated in this paper was further confirmed as having been reported as a HAB species in a previous study, with a certain reference (Table 1).

The richness indices (the number of algal species) and relative abundance (the percentage of each species in a sample) of the samples were calculated at the phylum, genus, and species level using Python (https://www.python.org/ (accessed on 14 July 2022)) scripts. The alpha diversity indices of algae were analyzed using the R package vegan [25], including richness, Pielou index [26], Shannon diversity [27], and Simpson diversity [28], and all of the indices were drawn with Prism 8 (version 8.0.2, GraphPad Software, San Diego, CA, USA). The pie chart, histogram figures, and line charts were drawn using Prism 8 (version 8.0.2, GraphPad Software, San Diego, CA, USA) and Adobe Illustrator CS6 (Adobe, San Jose, CA, USA). The maps of sampling sites, geographic distribution of HAB species, and environmental factors were plotted with Surfer 16 (version 16.0.330, Golden Software, Golden, CO, USA). The Upset diagram was drawn with the R package UpSetR [29]. A Mantel test between the environmental factors and five dominant HAB species was carried out by R package ggcor [30], which was visualized by R package ggplot2 [31].

## 3. Results

### 3.1. Composition and Alpha Diversity of Algae in QCS

**Figure 2:**
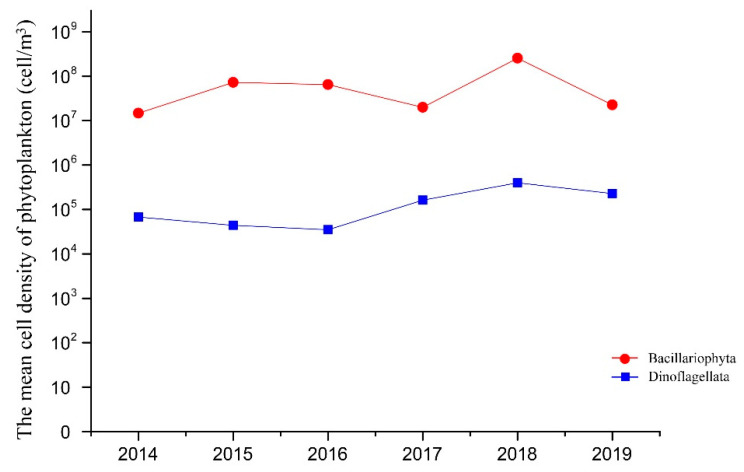
Interannual variation of dominant phytoplankton in the Qinhuangdao coastal sea from 2014 to 2019.

In the summers of 2014 to 2019, a total of 122 seawater fixed samples were collected in the QCS using a phytoplankton net. To investigate the interannual variation of algae in the QCS, the mean cell densities of Bacillariophyta and Dinoflagellata were calculated from 2014 to 2019 (Figure 2), and the cell density of Bacillariophyta (no less than 10^7^ cell/m^3^) was much higher than Dinoflagellata (no less than 10^4^ cell/m^3^) during this period. The population density of Bacillariophyta fluctuated slightly from 2014 to 2019, whereas the Dinoflagellata population density remarkably increased after 2016 (*p* < 0.05).

**Figure 3:**
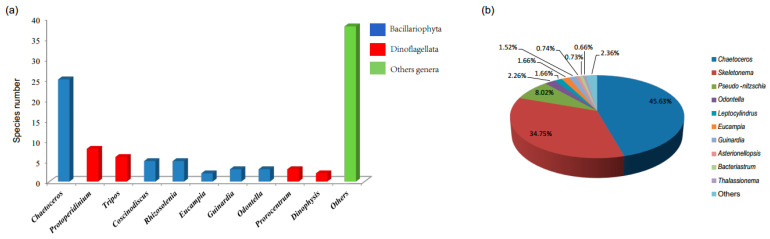
Phytoplankton richness and relative abundance at the genus level in the Qinhuangdao coastal sea. (**a**) Richness of phytoplankton at the genus level; (**b**) relative abundance of phytoplankton at the genus level.

In this project, we identified 100 unique algal species from 42 genera (8 classes of 3 phyla), after screening the 5 genera that could not be identified at the species level (Table S2). The 100 algal species could be categorized into three phyla/divisions, including Bacillariophyta (72 species), Dinoflagellata (26 species), and Ochrophyta (2 species). The relative abundance of Bacillariophyta was the highest (99.82%), much higher than that of Dinoflagellata (0.17%) and Ochrophyta (0.01%). At the genus level, the richness of *Chaetoceros* was the highest (25 species), followed by *Protoperidinium* (8 species) and *Tripos* (6 species) (Figure 3a). As for the top ten genera, there were six genera of Bacillariophyta and four of Dinoflagellata. Moreover, the *Chaetoceros* had the highest relative abundance (45.63%) (Figure 3b), followed by *Skeletonema* (34.75%) and *Pseudo-nitzschia* (8.02%). Overall, Bacillariophyta (primarily *Chaetoceros*) was the most dominant phylum in this region.

**Figure 4:**
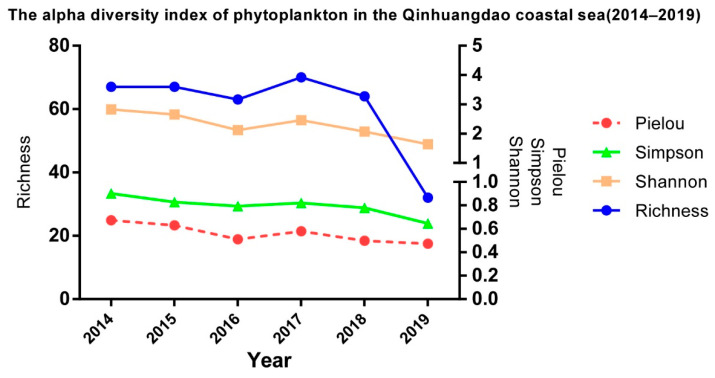
Alpha diversity of phytoplankton in the Qinhuangdao coastal sea in summers of 2014–2019.

Alpha diversity in the QCS was calculated using phytoplankton population density (Figure 4), including the species identified at the species and genus levels. The phytoplankton richness in the QCS was generally constant from 2014 to 2018 (63–70 species), but nearly halved in 2019 (32 species) due to a reduction in sampling sites. Meanwhile, the Shannon, Simpson, and Pielou indices differed, but were not statistically significant, with values decreasing slightly from 2014 to 2019.

### 3.2. Biodiversity and Distribution Patterns of HAB Species

Among the 100 algae species detected in the QCS, 59 were annotated as HAB species (Table 1), including 7 toxic HAB species. Of the 59 HAB species, 39, 18, and 2 species belonged to Bacillariophyta, Dinoflagellata, and Ochrophyta, respectively. The 59 HAB species belonged to 30 genera of 7 classes, and the biodiversity of *Chaetoceros* (11 HAB species) was the highest, followed by *Protoperidinium* (5 HAB species) and *Coscinodiscus* (4 HAB species).

**Figure 5:**
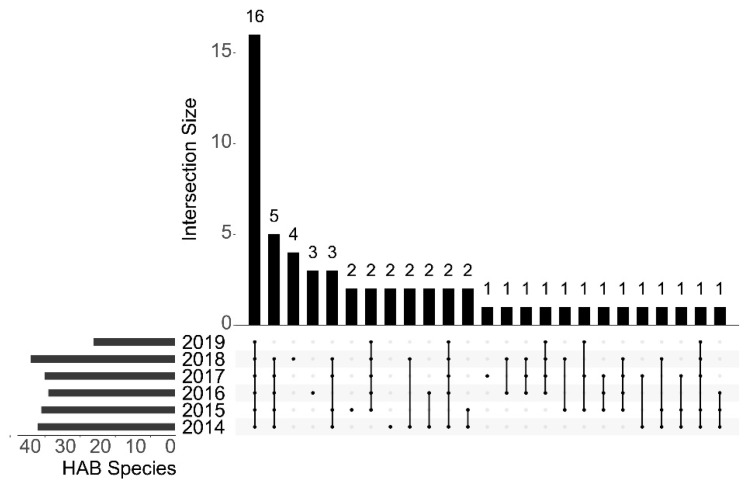
Upset diagram of HAB species in the Qinhuangdao coastal sea in the period of 2014–2019.

**Figure 6:**
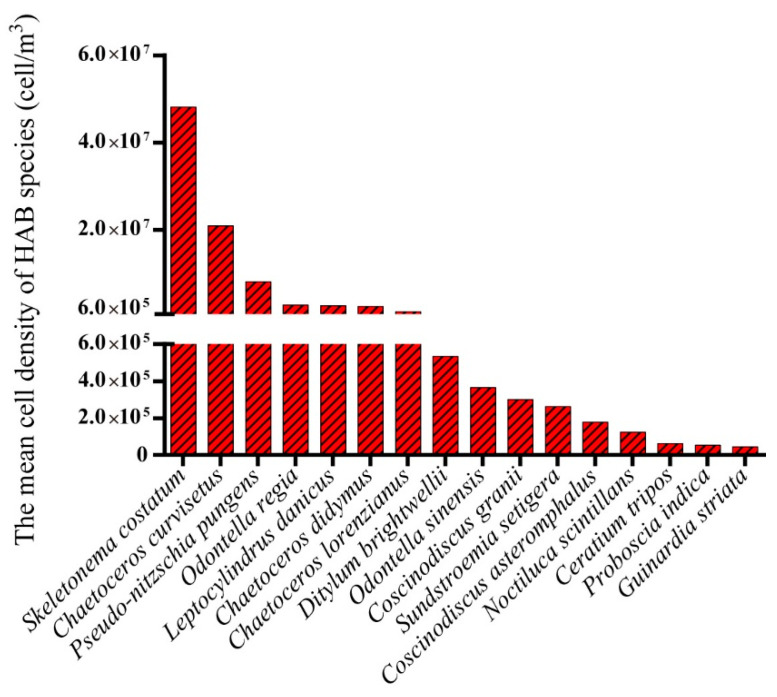
Mean cell density of 16 HAB species detected in all the surveys in the Qinhuangdao coastal sea.

There were 42 HAB species discovered in 2018, slightly more than the 37–40 species found from 2014 to 2017, and significantly more than 23 species discovered in 2019 (Figure 5), despite the fact that there were only 2 sampling sites in 2019. Through contrastive analysis, 16 HAB species (2 phyla, 5 classes, and 12 genera) were detected each year (Figure 6), including *Skeletonema costatum*, *Chaetoceros curvisetus*, *Noctiluca scintillans,* and *Ceratium tripos*. Of the 16 HAB species, Bacillariophyta (14 species) was much more common than Dinoflagellata (2 species). At the genus level, *Chaetoceros* (3 species) was highest, followed by *Coscinodiscus* (2 species) and *Odontella* (2 species). We calculated the average population density of the 16 HAB species during the study periods (Figure 6). The results showed that *Skeletonema costatum* had the highest density (4.82 × 10^7^ cell/m^3^), followed by the *Chaetoceros curvisetus* (2.09 × 10^7^ cell/m^3^) and *Pseudo-nitzschia pungens* (8.02 × 10^6^ cell/m^3^), which were significantly higher than the two Dinoflagellata HAB species, *Noctiluca scintillans* (1.24 × 10^5^ cell/m^3^) and *Ceratium tripos* (6.18 × 10^4^ cell/m^3^).

**Figure 7:**
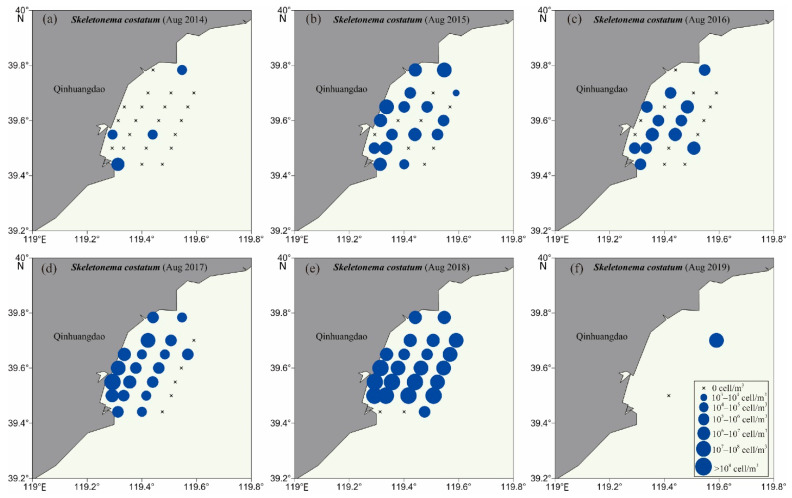
Distribution of *Skeletonema costatum* in the Qinhuangdao coastal sea at the time of six voyages, i.e., (**a**): 2014, (**b**): 2015, (**c**): 2016, (**d**): 2017, (**e**): 2018, (**f**): 2019.

**Figure 8:**
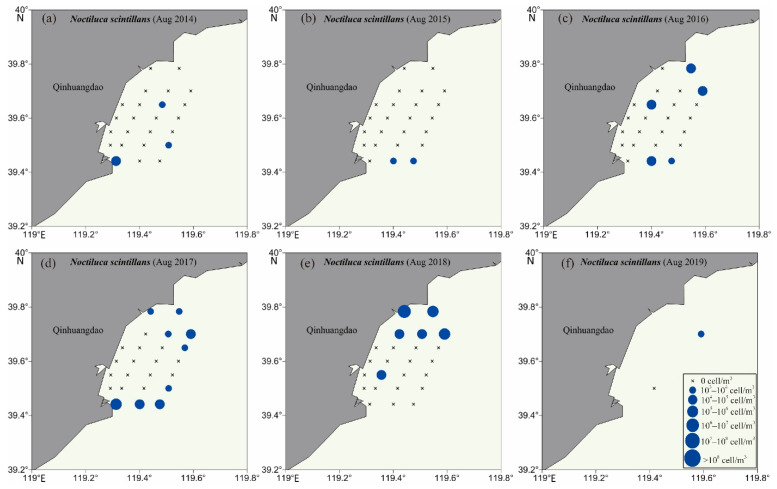
Distribution of *Noctiluca scintillans* in the Qinhuangdao coastal sea at the time of six voyages, i.e., (**a**): 2014, (**b**): 2015, (**c**): 2016, (**d**): 2017, (**e**): 2018, (**f**): 2019.

Four HAB species were chosen as examples to further investigate the interannual variation and geographical distribution of HAB species in the QCS, including two Bacillariophyta species (*Skeletonema costatum* and *Chaetoceros curvisetus*) and two Dinoflagellata species (*Noctiluca scintillans* and *Ceratium tripos*). The population density of *Skeletonema costatum* was relatively high at inshore sites of each sampling voyage (Figure 7), and the distribution range of the species gradually expanded from 4 sites (in 2014) to 21 sites (in 2018). However, *Chaetoceros curvisetus* was continuously distributed at most sampling sites of the QCS in summers of 2014–2019 with high population density (Figure S2). *Noctiluca scintillans* was detected at three sites in 2014 and two sites in 2015, and gradually expanded to nine sites in 2017 and six sites in 2018; meanwhile, the population density also increased (Figure 8). Different from the above three HAB species, *Ceratium tripos* was widely distributed at the most sites in 2014, 2017, and 2018, but only at half of the sites in 2015 and five sites in 2016 (Figure S3). The four HAB species were widely distributed in the surface seawaters of the QCS in the summers, and showed unique geographical distribution patterns in different years.

**Figure 9:**
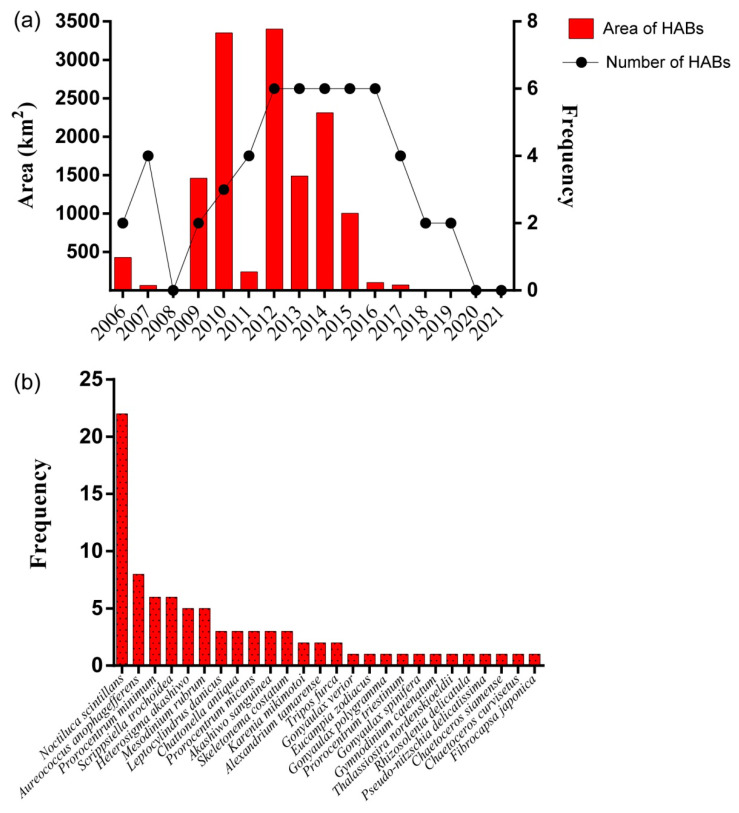
The interannual variation of HABs in the Qinhuangdao coastal sea from 2006 to 2021. (**a**) Area and frequency of HABs from 2006 to 2021; (**b**) frequency of 26 HAB species in the Qinhuangdao coastal sea.

By aggregating environment bulletins and historical literature [32,33,34], we had counted 53 HABs in the QCS during 2006–2021 over at least 13,935 km^2^ (Figure 9a), including 22 toxic HABs caused by *Prorocentrum minimum*, *Heterosigma akashiwo*, *Akashiwo sanguinea*, *Karenia mikimotoi,* etc. (Figure 9b). In total, 26 HAB species were detected in these HABs (Figure 9b), including 8 toxic HAB species. *Noctiluca scintillans* was the most common HAB species (22 occurrences) in the QCS in 2006–2021, followed by the *Aureococcus anophagefferens* (8 occurrences) and *Prorocentrum minimum* (6 occurrences); notably, there were 6 toxic species in the top 14 dominant blooming species (Figure 9b). Since 2016, a drastic reduction in the frequency and area of HABs in QCS was only observed for HABs with large areas, rather than small-size HABs.

**Figure 10:**
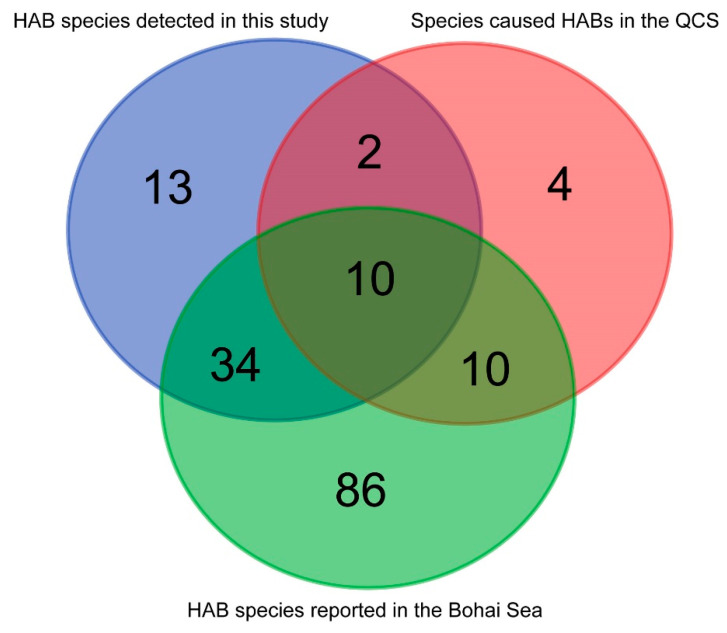
Venn analysis of HAB species in this study with the historical data of the Bohai Sea.

In total, 73 HAB species have been detected in the QCS in the past 16 years, including 59 species identified in this study and47 species (64.38%) that had never caused HABs in the QCS (Figure 10). In comparison to historical surveys conducted in the Bohai Sea between 1957 and 2019 [8], 44 HAB species (74.58%) detected in this study had been reported (Figure 10); nevertheless, 13 HAB species were the first to be recorded in the Bohai Sea.

### 3.3. Impact of Environmental Factors on Main HAB Species

There were no significant differences regarding environmental factors between different sampling expeditions, and the main reason is that the samples were collected during same period (August) in each year. However, the distribution of five environmental factors in the surface seawaters showed unique geographical patterns in the summers of 2014–2018 in the QCS (Figure S1). In 2014–2018, the average temperature ranged from 25.83 to 28.1 °C. The salinity in the QCS ranged from 29.94 to 31.35, with values gradually increasing as the distance from the sampling site to the shore increased. On the contrary, owing to nutrient inputs from rivers, the values of chlorophyll-a, DIN, and DIP at the inshore sites were generally higher than those at the distant sites. Notably, the average concentration of chlorophyll-a was very high in the surface seawaters in 2018 (13.53 μg/L), even reaching 43.3 μg/L at the BY21 site, while *Pseudo-nitzschia delicatissima* was the first domain species at the BY21 site (1.42 × 10^6^ cell/m^3^), and *Skeletonema costatum* was the first domain species at most sites, even reaching 1.28 × 10^9^ cell/m^3^ at the BY07 site.

To explore the impact of environmental factors on HAB species, the correlations of environmental factors with five dominant HAB species in 2014–2018 were calculated (Figure S4). The DIN had significant correlations (*p* < 0.05, |r| > 0.5) with *Noctiluca scintillans* and *Skeletonema costatum* in 2014, and *Noctiluca scintillans* had significant correlations with DIN (*p* < 0.05, |r| > 0.3) and temperature (*p* < 0.01, |r| > 0.5) in 2015. In 2016, chlorophyll-a had significant correlations with *Chaetoceros curvisetus* (*p* < 0.05, |r| > 0.3) and *Pseudo-nitzschia pungens* (*p* < 0.05, |r| > 0.8). In 2018, *Chaetoceros curvisetus* and *Ceratium tripos* both had significant correlations (*p* < 0.05, |r| > 0.3) with salinity. However, there was no significant correlation of environmental factors with the five HAB species in 2017.

## 4. Discussion

The QCS is well known for the frequent occurrence of HABs in the Bohai Sea [34,35], but there were no systematic studies on HAB species diversity in the area. In this study, we analyzed the biodiversity and interannual variation of algal species in the QCS and tested the hypothesis that the high occurrences of diverse HABs may be caused by the presence of a large number of HAB species in this region. From 2014 to 2019, a large number of HAB species were detected in the QCS, including some species that had never been reported in the Bohai Sea, which may explain why HABs were so prevalent in the QCS.

### 4.1. Interannual Variation of Algae in the Qinhuangdao Coastal Sea

In summers of 2014–2019, the density of Bacillariophyta (>10^7^ cell/m^3^) was much higher than Dinoflagellata (>10^4^ cell/m^3^) in the QCS, which is consistent with previous studies using the Operational Taxonomy Units (OTUs)-based metabarcoding analysis of samples collected from the QCS in 2013–2014 [35]. The result of those studies indicated that the dominant phytoplankton population in the QCS showed obvious seasonal variations from micro-chain diatoms to dinoflagellates in spring, to small diatoms in summer, and then to micro-chain diatoms in fall and winter. In that study, 123 phytoplankton species from 4 phyla were identified via microscopy in 2013–2014 [35], including 87 diatom species and 33 dinoflagellate species. Therefore, over the whole year, diatoms were the dominant algae in the QCS during most sampling periods, with high population density and biodiversity.

It is remarkable that the mean density of Dinoflagellata gradually increased in the QCS after 2016 (Figure 2), which is not an isolated case in the coastal sea of China. Some researchers found that the dominant blooming algae species in the Changjiang River estuary was shifting from diatoms to dinoflagellates, partly due to nutrient changes in this region [19,36,37]. Furthermore, there were obvious interannual variations in the blooming dinoflagellate species, as well as an increase in toxic algae species [38]. By the end of the 21st century, the biomass of diatoms in the near-shore sea of the East China Sea may decrease by 19%, while the biomass of dinoflagellates may increase by 60% [39,40]. Xiao et al. (2019) predicted that the relative abundance of Dinoflagellates, Prochlorococcus, Synechococcus, and Haptophytes_6 in the South China Sea would increase with global warming, and that the temperature and light intensity of the surface mixed layer would increase [41]. Therefore, the increasing trend of dinoflagellates may become common in the coastal waters of China in the future, which is worthy of continuous monitoring.

Since the 1930s, scientists have used morphology-based methods to investigate the biodiversity of phytoplankton in the Bohai Sea, collecting extensive and valuable historical data [8]. In this study, 100 algae species of 3 phyla were identified using the morphology-based method, but this number might be underestimating the true biodiversity of algae in the QCS. Aside from the insufficient sampling seasons and sites, the identification method of algae species was the major challenge. There is no denying that the morphological approach has some limitations in identifying algae species. For example, it is difficult to identify a microalgae species with a small diameter using a traditional optical microscope [42,43], especially one less than 3 μm, such as *Aureococcus anophagefferens* and *Phaeocystis globosa*. In addition, some fragile algae species are difficult to collect, or even damaged or changed in morphology by traditional sampling methods [44], such as the *Heterosigma akashiwo* and *Akashiwo sanguinea* [22,45]. In addition, some algae species have high genetic diversity, or contain a large number of cryptic species. The algal strains with different genetic diversity and cryptic species may exhibit varying physiological characteristics and distribution patterns, contributing differently to the formation of HABs [46,47,48] such as the toxic *Alexandrium tamarense* species complex [46]. Under different temperature and salinity conditions, the growth and reproduction capacity of *Alexandrium tamarense* strains isolated from Changjiang Estuary were clearly greater than those of strains isolated from Minjiang Estuary and Beibu Gulf, indicating that the cryptic species of *Alexandrium tamarense,* with different distribution patterns and physiological adaptation characteristics, has a different ability to adapt to the marine environment [49]. Afterwards, *Alexandrium tamarense* was found to be a species complex with multiple distinctive species, including *Alexandrium tamarense*, *Alexandrium fundyense*, *Alexandrium australiense*, *Alexandrium mediterraneum,* and *Alexandrium pacifcum* [46]. In the QCS, red tides of *Alexandrium tamarense* occurred in 2016 and 2017 [34], and *Alexandrium catenella* bloomed in 2019–2021, often with a small sea area, so these HABs were usually overlooked. These toxic species have a huge impact on aquaculture and human health in the QCS; thus, it is necessary to distinguish different species with high genetic diversity and cryptic species accurately and timely.

Morphological identification of algae species is a common method which is relatively simple and quick, but it requires the researcher to have sufficient experience and accumulation of knowledge. With the development of sequencing technology and the reduction in sequencing cost, an environmental DNA-based metabarcoding approach has been widely used in marine biodiversity studies [50]. The metabarcoding approach is an important complement to traditional observational methods. With the perfection of technology and standardization of sampling methods, it could even bring global biomonitoring to a level of observation similar to that of ocean physics and biogeochemistry [51]. In recent decades, metabarcoding methods, particularly Amplicon Sequence Variants (ASVs)-based metabarcoding analysis methods with the Divisive Amplicon Denoising Algorithm (DADA2), have been widely used to investigate the composition and distribution of plankton species [52,53,54]. Notably, the ASV-based methods do not rely on reference databases or similarity thresholds which can be used to compare the species composition of samples from different studies [50,53,54]. In summer of 2019, 74 potential HAB species were identified in the Bohai Sea through sequencing and analyzing the 18S rDNA V4 region, highlighting the sensitivity and power of the ASVs-based metabarcoding method in detecting HAB species [12]. However, the metabarcoding method still has some drawbacks. Owing to the limitations of the Molecular Marker Database and the resolution of common molecular markers, a large number of sequencing results cannot be annotated [55], resulting in the loss of some critical information about the sample. With the continuous updates to the reference databases, the development of high-resolution molecular markers, and the advancement of sequencing technology, identification of species in the marine plankton community will become more accurate and efficient in future studies.

### 4.2. High Biodiversity of HAB Species in the Qinhuangdao Coastal Sea

In this study, more than half of the identified algal species were HAB species (59 out of 100), of which 16 HAB species were detected in all 6 years in the QCS, including *Skeletonema costatum*, *Chaetoceros curvisetus*, *Noctiluca scintillans,* and *Ceratium tripos*. Overall, the geographic distribution and population density of the four dominant HAB species increased from 2014 to 2019, presenting a high risk of algal bloom outbreak, especially the two dinoflagellate species *Noctiluca scintillans* and *Ceratium tripos*, both of which bloomed in the summers of 2017 and 2022 in the QCS [34]. In 2013, twelve surveys were conducted from April to October near the coast of Qinhuangdao, in which the abundance of Dinophyceae was relatively high [56]. In our previous study, we found that Dinoflagellata was the dominant phylum of phytoplankton in the Bohai Sea from July to August in 2019, and 43 Dinoflagellata HAB species were annotated using the ASV-based metabarcoding method [12]. However, due to the lack of long-term data from different years, further analysis is needed to determine whether the gradual transformation of dominant phytoplankton species is changing in local areas, such as offshore areas, estuaries, and bays, or to investigate the evolution of the communities in the overall environment of the Bohai Sea.

Of the 59 HAB species, 47 species (64.38%) had never caused HABs in the QCS in the past 16 years, but nearly three quarters of these HAB species were reported in the Bohai Sea (Figure 10) and were well known for causing HABs elsewhere in the world, including *Pseudo-nitzschia pungens*, *Odontella regia*, *Chaetoceros lorenzianus,* and *Chaetoceros didymus*. These four HAB species were detected, in all six voyages (2014–2019), to have high population density (more than 10^6^ cell/m^3^) in the QCS. In particular, the *Pseudo-nitzschia pungens* even reached 5.22 × 10^8^ cell/m^3^, with the highest concentration of chlorophyll-a (21.6 μg/L) at the BY23 site in 2016. Thus, we speculate that some algae species might have caused small-sized HABs in the QCS, and these HAB species had a potential risk to develop into large-sized HABs, especially in suitable environmental conditions. Moreover, 13 HAB species, including *Gyrodinium spirale* and *Protoperidinium conicum*, were reported in the Bohai Sea for the first time. Some of these species may even have caused HABs in the Bohai Sea. Thus, it is even more important to focus on future potential ecology disasters caused by these HAB species.

In the long term, the HAB species in Chinese coastal seawaters are characterized by diversification, miniaturization, and harmfulness [4]. In this study, seven toxic HAB species were detected in the QCS, including two *Pseudo-nitzschia* species that produce domoic acid (DA). The *Pseudo-nitzschia delicatissima* bloomed in August of 2007 in the QCS [34] and widely distributed in the QCS in 2014–2018, even reaching 1.37 × 10^7^ cell/m^3^ at the BY08 site in 2018. Although it has been shown to be toxic in culture [57], the toxic strains of *Pseudo-nitzschia delicatissima* have never been detected in the coastal sea of China. The *Pseudo-nitzschia pungens*, a dominant HAB species in the QCS, not only frequently caused disasters in the China, but was also reported in Europe, North America, South America, and Oceania. It has high genetic diversity, and some strains can produce DA, which is harmful to animals and humans [57,58]. Until now, the toxic strains of *Pseudo-nitzschia pungens* only have been reported in the New Zealand and the west coast of the USA [59]; thus, the distribution of toxic strains and the genetic diversity of this species in the coastal seawaters of China deserves further research.

Environmental factors play an important role in the distribution and community structure of HAB species, and even in the generation and elimination of HABs [1,60]. In this study, the five environmental factors showed no significant changes in the summers from 2014 to 2019, but there were differences in geographical distribution (Figure S1). Meanwhile, the DIN had significant correlations with *Noctiluca scintillans* in 2014–2015, and salinity had significant correlations with *Chaetoceros curvisetus* and *Ceratium tripos* in 2018 (Figure S4d). The Qinhaungdao city has many active rivers, including the Yanghe River, Daihe River, Tanghe River, and Shihe River, carrying large organic material and dissolved trace elements into the Liaodong Bay of the Bohai Sea. Of these, the DIN [13] is especially significant, which may influence the distribution of phytoplankton and even the process of development of HABs in the area, thus reducing the input of terrigenous nutrients which are essential for the prevention of HABs in the region. Diatoms and dinoflagellates are two dominant phytoplankton groups in the marine ecology system, and both are the groups with the highest occurrence rate of HABs. Dinoflagellate algal blooms are more harmful than diatom algal blooms [1,61]. Therefore, the response of these two phytoplankton groups to future environmental changes is related to the health of the offshore ecosystem [39].

## 5. Conclusions

Through detecting and identifying algal species in samples collected in the QCS from 2014 to 2019, this study has enriched our understanding of the community and interannual variation of HAB species in the QCS. We have identified 100 algae species in the area, including 59 HAB species, which may explain why HABs frequently occurred in the QCS. Bacillariophyta was the most dominant phylum of the phytoplankton community during the voyage surveys in 2014–2019, but the population density of Dinoflagellata has gradually increased since 2016. Similarly, the distribution and population density of four dominant HAB species also increased from 2014 to 2019 in the QCS.

Above all, high biodiversity of HAB species existed in the QCS, with a high risk of HABs. Meanwhile, the distribution area and population density of HAB species increased in recent years. In the next few years, the monitoring of dinoflagellate HABs needs to be strengthened, and molecular marker technology is an important support method for traditional morphological identification.

## Figures and Tables

**Table 1:** List of 59 HAB species identified in this study.

NO	Species	Phylum	Class	Toxicology	Year	HAB Evidence Reference
1	*Akashiwo sanguinea*	Dinoflagellata	Dinophyceae	Ichthyotoxic	2014/2016/2018	[22]
2	*Asterionellopsis glacialis*	Bacillariophyta	Bacillariophyceae	NO	2015–2019	[22]
3	*Bacillaria paxillifera*	Bacillariophyta	Bacillariophyceae	NO	2014–2015	[22]
4	*Cerataulina pelagica*	Bacillariophyta	Mediophyceae	NO	2014–2018	[8]
5	*Ceratium tripos*	Dinoflagellata	Dinophyceae	NO	2014–2019	[8]
6	*Chaetoceros affinis*	Bacillariophyta	Mediophyceae	NO	2014–2015/2017–2019	[8]
7	*Chaetoceros compressus*	Bacillariophyta	Mediophyceae	NO	2014–2018	[8]
8	*Chaetoceros curvisetus*	Bacillariophyta	Mediophyceae	NO	2014–2019	[8]
9	*Chaetoceros debilis*	Bacillariophyta	Mediophyceae	NO	2015/2018	[8]
10	*Chaetoceros diadema*	Bacillariophyta	Mediophyceae	NO	2014	[8]
11	*Chaetoceros didymus*	Bacillariophyta	Mediophyceae	NO	2014–2019	[8]
12	*Chaetoceros lorenzianus*	Bacillariophyta	Mediophyceae	NO	2014–2019	[8]
13	*Chaetoceros paradoxus*	Bacillariophyta	Mediophyceae	NO	2014–2018	[17]
14	*Chaetoceros peruvianus*	Bacillariophyta	Mediophyceae	NO	2018	[22]
15	*Chaetoceros siamense*	Bacillariophyta	Mediophyceae	NO	2015–2019	[8]
16	*Chaetoceros socialis*	Bacillariophyta	Mediophyceae	NO	2014–2015/2018	[8]
17	*Chattonella marina*	Ochrophyta	Raphidophyceae	Ichthyotoxic	2018	[8]
18	*Coscinodiscus asteromphalus*	Bacillariophyta	Coscinodiscophyceae	NO	2014–2019	[8]
19	*Coscinodiscus granii*	Bacillariophyta	Coscinodiscophyceae	NO	2014–2019	[8]
20	*Coscinodiscus radiatus*	Bacillariophyta	Coscinodiscophyceae	NO	2014	[8]
21	*Coscinodiscus wailesii*	Bacillariophyta	Coscinodiscophyceae	NO	2014/2016–2019	[8]
22	*Dictyocha fibula*	Ochrophyta	Dictyochophyceae	NO	2014–2015/2017–2018	[22]
23	*Dinophysis acuminata*	Dinoflagellata	Dinophyceae	Okadaic acid (OA) and Dinophysis toxin (DTX)	2014/2018	[8]
24	*Ditylum brightwellii*	Bacillariophyta	Mediophyceae	NO	2014–2019	[22]
25	*Eucampia cornuta*	Bacillariophyta	Mediophyceae	NO	2016–2018	[8]
26	*Eucampia zodiacus*	Bacillariophyta	Mediophyceae	NO	2014–2016	[8]
27	*Guinardia delicatula*	Bacillariophyta	Coscinodiscophyceae	NO	2016/2018	[8]
28	*Guinardia flaccida*	Bacillariophyta	Coscinodiscophyceae	NO	2015–2017	[8]
29	*Guinardia striata*	Bacillariophyta	Coscinodiscophyceae	NO	2014–2019	[22]
30	*Gymnodinium catenatum*	Dinoflagellata	Dinophyceae	Paralytic shellfish toxins (PSTs)	2016	[17]
31	*Gyrodinium spirale*	Dinoflagellata	Dinophyceae	NO	2014–2015/2017–2018	[22]
32	*Leptocylindrus danicus*	Bacillariophyta	Mediophyceae	NO	2014–2019	[17]
33	*Leptocylindrus minimus*	Bacillariophyta	Mediophyceae	NO	2016	[23]
34	*Nitzschia closterium*	Bacillariophyta	Bacillariophyceae	NO	2014–2015/2017–2018	[8]
35	*Noctiluca scintillans*	Dinoflagellata	Noctilucaceae	NO	2014–2019	[22]
36	*Odontella mobiliensis*	Bacillariophyta	Mediophyceae	NO	2018	[8]
37	*Odontella regia*	Bacillariophyta	Mediophyceae	NO	2014–2019	[8]
38	*Odontella sinensis*	Bacillariophyta	Mediophyceae	NO	2014–2019	[22]
39	*Proboscia alata*	Bacillariophyta	Mediophyceae	NO	2015/2017/2019	[22]
40	*Proboscia indica*	Bacillariophyta	Mediophyceae	NO	2014–2019	[22]
41	*Prorocentrum micans*	Dinoflagellata	Dinophyceae	NO	2018	[8]
42	*Prorocentrum minimum*	Dinoflagellata	Dinophyceae	Tetrodotoxins (TTXs)	2014/2016	[8]
43	*Prorocentrum triestinum*	Dinoflagellata	Dinophyceae	NO	2014–2015/2017	[22]
44	*Protoperidinium bipes*	Dinoflagellata	Dinophyceae	NO	2014/2016	[24]
45	*Protoperidinium conicum*	Dinoflagellata	Dinophyceae	NO	2016–2019	[22]
46	*Protoperidinium elegans*	Dinoflagellata	Dinophyceae	NO	2014–2015	[22]
47	*Protoperidinium pellucidum*	Dinoflagellata	Dinophyceae	NO	2014/2017	[22]
48	*Protoperidinium pentagonum*	Dinoflagellata	Dinophyceae	NO	2017	[22]
49	*Pseudo-nitzschia delicatissima*	Bacillariophyta	Bacillariophyceae	Domoic acid (DA)	2014–2018	[8]
50	*Pseudo-nitzschia pungens*	Bacillariophyta	Bacillariophyceae	Domoic acid (DA)	2014–2019	[22]
51	*Pyrophacus steinii*	Dinoflagellata	Dinophyceae	NO	2015–2018	[22]
52	*Rhizosolenia styliformis*	Bacillariophyta	Coscinodiscophyceae	NO	2015	[22]
53	*Skeletonema costatum*	Bacillariophyta	Mediophyceae	NO	2014–2019	[22]
54	*Stephanopyxis palmeriana*	Bacillariophyta	Coscinodiscophyceae	NO	2014/2016–2019	[22]
55	*Sundstroemia setigera*	Bacillariophyta	Coscinodiscophyceae	NO	2014–2019	[8]
56	*Thalassionema nitzschioides*	Bacillariophyta	Bacillariophyceae	NO	2014/2018	[22]
57	*Tripos breve*	Dinoflagellata	Dinophyceae	NO	2016	[22]
58	*Tripos fusus*	Dinoflagellata	Dinophyceae	NO	2014–2018	[8]
59	*Tripos massiliensis*	Dinoflagellata	Dinophyceae	NO	2015	[22]

## Data Availability

Data are contained within the article or Supplementary Material.

## Supplementary Materials

The following supporting information can be downloaded at: https://www.mdpi.com/article/10.3390/life13010192/s1, There are two tables as Supplementary Materials of the manuscript, and the captions for the Supplementary Tables are also included. Table S1: Sampling sites and environmental factors in the Qinhuangdao coastal sea in summers of 2014–2019. Table S2: List of 100 algal species identified in this study. There are 4 figures as Supplementary Materials of the manuscript, and the captions for the Supplementary Figures are also included. Figure S1: Distribution of environmental factors in the surface seawater of the Qinhuangdao coastal sea in summers of 2014–2018. Figure S2: Distribution of *Chaetoceros curvisetus* in the Qinhuangdao coastal sea in six voyages, i.e., (a): 2014, (b): 2015, (c): 2016, (d): 2017, (e): 2018, (f): 2019. Figure S3: Distribution of *Ceratium tripos* in the Qinhuangdao coastal sea in six voyages, i.e., (a): 2014, (b): 2015, (c): 2016, (d): 2017, (e): 2018, (f): 2019. Figure S4: Correlation analysis of environmental factors with five main HAB species in the Qinhuangdao coastal sea in 2014 (a), 2015 (b), 2016 (c), and 2018 (d).
