# Supplementary material for: Biodiversity and Interannual Variation of Harmful Algal Bloom Species in the Coastal Sea of Qinhuangdao, China
Source: Life (Basel). 2023 Jan 9;13(1):192. doi: 10.3390/life13010192 (PMC9867081; doi:10.3390/life13010192)
Supplement: Supplementary file 1 [file life-13-00192-s001.zip › Supplementary Tables.pdf]

There are two tables as supplementary materials of the manuscript, and with the captions for supplementary tables.

Table S1 Sampling sites and environmental factors in the Qinhuangdao coastal sea in summers of 2014–2019

Longitude	Latitude	Sampling sites	Date	Temperature (℃)	Salinity (PSU)	Chlorophyll-a (μg/L)	DIN (mg/L)	DIP (mg/L)
119.29-119.59°E	39.44-39.78°N	24 (BY01-BY24)	August 6-18, 2014	25.83±0.48	29.94±0.72	4.74±4.00	0.1708±0.0423	0.0075±0.0021
119.29-119.59°E	39.44-39.78°N	24 (BY01-BY24)	August 7-23, 2015	28.10±1.10	30.80±0.46	5.90±5.11	0.1417±0.0319	0.0075±0.0035
119.29-119.59°E	39.44-39.78°N	24 (BY01-BY24)	August 10-29, 2016	27.00±0.78	30.47±0.68	6.89±3.79	0.1503±0.081	0.0021±0.0017
119.29-119.59°E	39.44-39.78°N	24 (BY01-BY24)	August 9-26, 2017	27.72±0.58	31.36±0.44	3.42±2.04	0.0631±0.0296	0.0045±0.0034
119.29-119.59°E	39.44-39.78°N	24 (BY01-BY24)	August 2-26, 2018	26.66±0.44	30.14±1.43	12.88±9.61	0.0865±0.085	0.0077±0.0084
119.42-119.59°E	39.50-39.70°N	2 (BY06 and BY22)	August 8-19, 2019	27.30±0.50	31.62±0.47	3.84±2.48	0.1167±0.0665	0.0023±0.0003

Table S2 List of 100 algal species identified in this study

NO	Species	Phylum	Class
1	*Achnanthes brevipes*	Bacillariophyta	Bacillariophyceae
2	*Akashiwo sanguinea*	Dinoflagellata	Dinophyceae
3	*Asterionellopsis glacialis*	Bacillariophyta	Bacillariophyceae
4	*Bacillaria paxillifera*	Bacillariophyta	Bacillariophyceae
5	*Bacteriastrum hyalinum*	Bacillariophyta	Bacillariophyceae
6	*Biddulphia biddulphiana*	Mediophyceae	Bacillariophyceae
7	*Cerataulina pelagica*	Bacillariophyta	Mediophyceae
8	*Ceratium breve*	Dinoflagellata	Dinophyceae
9	*Ceratium furca*	Dinoflagellata	Dinophyceae
10	*Ceratium fusus*	Dinoflagellata	Dinophyceae
11	*Ceratium lineatum*	Dinoflagellata	Dinophyceae
12	*Ceratium massiliense*	Dinoflagellata	Dinophyceae
13	*Ceratium tripos*	Dinoflagellata	Dinophyceae
14	*Chaetoceros affinis*	Bacillariophyta	Mediophyceae
15	*Chaetoceros atlanticus var. atlanticus*	Bacillariophyta	Mediophyceae
16	*Chaetoceros castracanei*	Bacillariophyta	Mediophyceae
17	*Chaetoceros coarctatus*	Bacillariophyta	Mediophyceae
18	*Chaetoceros compressus*	Bacillariophyta	Mediophyceae
19	*Chaetoceros constrictus*	Bacillariophyta	Mediophyceae
20	*Chaetoceros crinitus*	Bacillariophyta	Mediophyceae
21	*Chaetoceros curvisetus*	Bacillariophyta	Mediophyceae
22	*Chaetoceros debilis*	Bacillariophyta	Mediophyceae
23	*Chaetoceros decipiens f. decipiens*	Bacillariophyta	Mediophyceae

24	*Chaetoceros decipiens f. singularis*	Bacillariophyta	Mediophyceae
25	*Chaetoceros densus*	Bacillariophyta	Mediophyceae
26	*Chaetoceros diadema*	Bacillariophyta	Mediophyceae
27	*Chaetoceros didymus*	Bacillariophyta	Mediophyceae
28	*Chaetoceros distans*	Bacillariophyta	Mediophyceae
29	*Chaetoceros knipowitschii*	Bacillariophyta	Mediophyceae
30	*Chaetoceros lorenzianus*	Bacillariophyta	Mediophyceae
31	*Chaetoceros nipponica*	Bacillariophyta	Mediophyceae
32	*Chaetoceros paradoxus*	Bacillariophyta	Mediophyceae
33	*Chaetoceros peruvianus*	Bacillariophyta	Mediophyceae
34	*Chaetoceros siamense*	Bacillariophyta	Mediophyceae
35	*Chaetoceros socialis*	Bacillariophyta	Mediophyceae
36	*Chaetoceros teres*	Bacillariophyta	Mediophyceae
37	*Chaetoceros tortissimus*	Bacillariophyta	Mediophyceae
38	*Chaetoceros vanheurcki*	Bacillariophyta	Mediophyceae
39	*Chattonella marina*	Ochrophyta	Raphidophyceae
40	*Corethron criophilum*	Bacillariophyta	Mediophyceae
41	*Coscinodiscus asteromphalus*	Bacillariophyta	Coscinodiscophyceae
42	*Coscinodiscus granii*	Bacillariophyta	Coscinodiscophyceae
43	*Coscinodiscus radiatus*	Bacillariophyta	Coscinodiscophyceae
44	*Coscinodiscus subtilis*	Bacillariophyta	Coscinodiscophyceae
45	*Coscinodiscus wailesii*	Bacillariophyta	Coscinodiscophyceae
46	*Dictyocha fibula*	Ochrophyta	Dictyochophyceae
47	*Dinophysis acuminata*	Dinoflagellata	Dinophyceae
48	*Dinophysis caudate*	Dinoflagellata	Dinophyceae
49	*Ditylum brightwellii*	Bacillariophyta	Mediophyceae

50	*Eucampia cornuta*	Bacillariophyta	Mediophyceae
51	*Eucampia zodiacus*	Bacillariophyta	Mediophyceae
52	*Guinardia delicatula*	Bacillariophyta	Coscinodiscophyceae
53	*Guinardia flaccida*	Bacillariophyta	Coscinodiscophyceae
54	*Guinardia striata*	Bacillariophyta	Coscinodiscophyceae
55	*Gymnodinium catenatum*	Dinoflagellata	Dinophyceae
56	*Gyrodinium spirale*	Dinoflagellata	Dinophyceae
57	*Helicotheca tamesis*	Bacillariophyta	Mediophyceae
58	*Helicotheca tamesis*	Bacillariophyta	Mediophyceae
59	*Hemiaulus sinensis*	Bacillariophyta	Mediophyceae
60	*Leptocylindrus danicus*	Bacillariophyta	Mediophyceae
61	*Leptocylindrus minimus*	Bacillariophyta	Mediophyceae
62	*Licmophora abbreviata*	Bacillariophyta	Bacillariophyceae
63	*Lithodesmium undulatum*	Bacillariophyta	Mediophyceae
64	*Melosira sulcata*	Bacillariophyta	Coscinodiscophyceae
65	*Nitzschia closterium*	Bacillariophyta	Bacillariophyceae
66	*Nitzschia lorenziana*	Bacillariophyta	Bacillariophyceae
67	*Noctiluca scintillans*	Dinoflagellata	Noctilucaceae
68	*Odontella mobiliensis*	Bacillariophyta	Mediophyceae
69	*Odontella regia*	Bacillariophyta	Mediophyceae
70	*Odontella sinensis*	Bacillariophyta	Mediophyceae
71	*Peridinium quinquecorne*	Dinoflagellata	Dinophyceae
72	*Pleurosigma pelagicum*	Bacillariophyta	Bacillariophyceae
73	*Prorocentrum micans*	Dinoflagellata	Dinophyceae
74	*Prorocentrum minimum*	Dinoflagellata	Dinophyceae
75	*Prorocentrum triestinum*	Dinoflagellata	Dinophyceae

76	*Protoperidinium bipes*	Dinoflagellata	Dinophyceae
77	*Protoperidinium conicum*	Dinoflagellata	Dinophyceae
78	*Protoperidinium elegans*	Dinoflagellata	Dinophyceae
79	*Protoperidinium grande*	Dinoflagellata	Dinophyceae
80	*Protoperidinium pallidum*	Dinoflagellata	Dinophyceae
81	*Protoperidinium pellucidum*	Dinoflagellata	Dinophyceae
82	*Protoperidinium pentagonum*	Dinoflagellata	Dinophyceae
83	*Protoperidinium venustum*	Dinoflagellata	Dinophyceae
84	*Pseudo-nitzschia delicatissima*	Bacillariophyta	Bacillariophyceae
85	*Pseudo-nitzschia pungens*	Bacillariophyta	Bacillariophyceae
86	*Pyrophacus steinii*	Dinoflagellata	Dinophyceae
87	*Rhizosolenia alata f. gracillima*	Bacillariophyta	Coscinodiscophyceae
88	*Rhizosolenia alata f. indica*	Bacillariophyta	Coscinodiscophyceae
89	*Rhizosolenia hyalina*	Bacillariophyta	Coscinodiscophyceae
90	*Rhizosolenia setigera*	Bacillariophyta	Coscinodiscophyceae
91	*Rhizosolenia styliformis*	Bacillariophyta	Coscinodiscophyceae
92	*Schrōderella delicatula f. schrōderi*	Bacillariophyta	Coscinodiscophycidae
93	*Scrippsiella trochoidea*	Dinoflagellata	Dinophyceae
94	*Skeletonema costatum*	Bacillariophyta	Mediophyceae
95	*Stephanopyxis palmeriana*	Bacillariophyta	Coscinodiscophyceae
96	*Stephanopyxis turris*	Bacillariophyta	Coscinodiscophyceae
97	*Thalassionema frauenfeldii*	Bacillariophyta	Bacillariophyceae
98	*Thalassionema nitzschioides*	Bacillariophyta	Bacillariophyceae
99	*Thalassiosira nordenskioldi*	Bacillariophyta	Bacillariophyceae
100	*Thalassiostra rotula*	Bacillariophyta	Bacillariophyceae
