# Supplementary figures and images for: Biodiversity and Interannual Variation of Harmful Algal Bloom Species in the Coastal Sea of Qinhuangdao, China
Source: Life (Basel). 2023 Jan 9;13(1):192. doi: 10.3390/life13010192 (PMC9867081; doi:10.3390/life13010192)

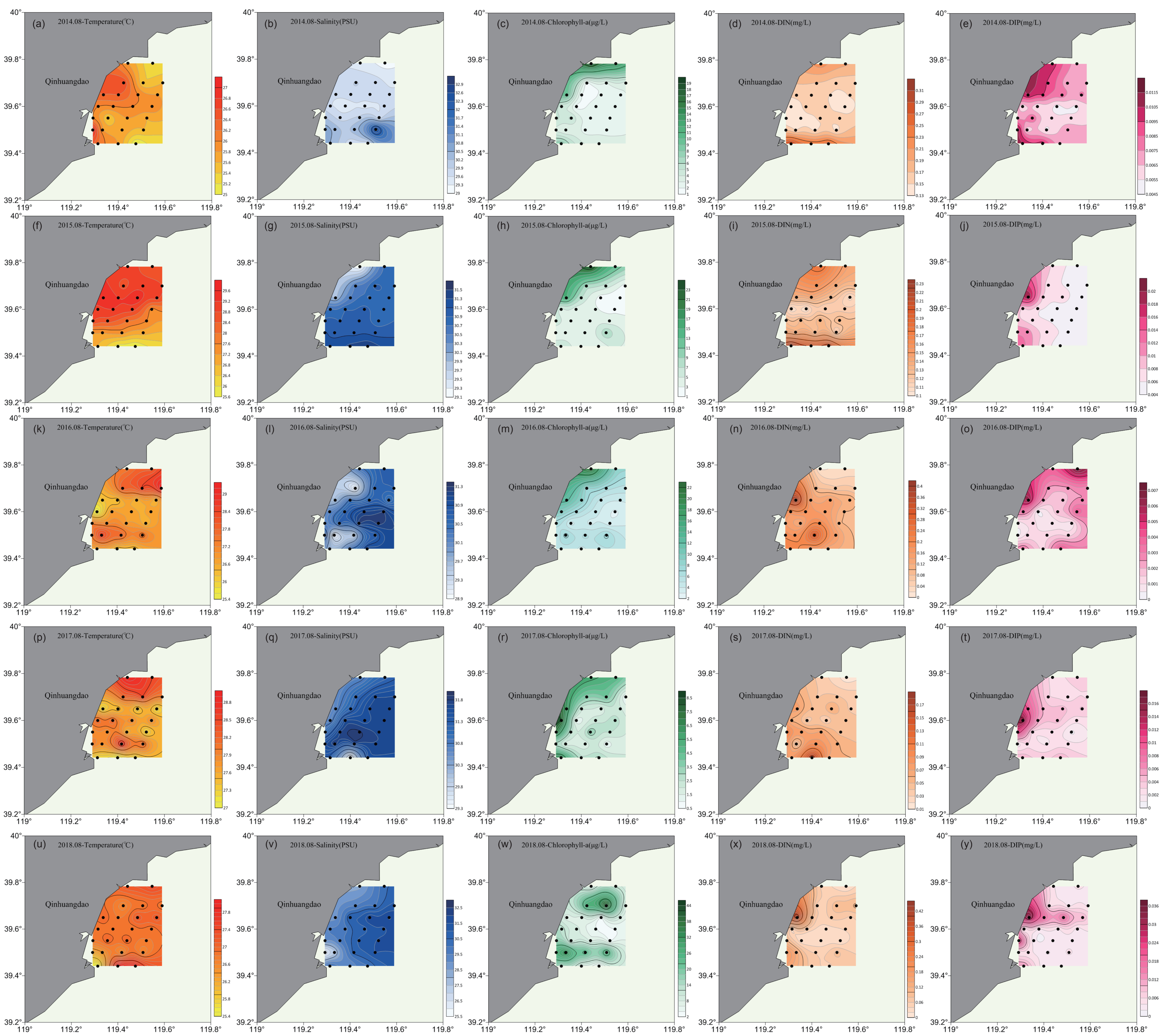

Supplement: Supplementary file 1 [file life-13-00192-s001.zip › Figure S1.pdf]

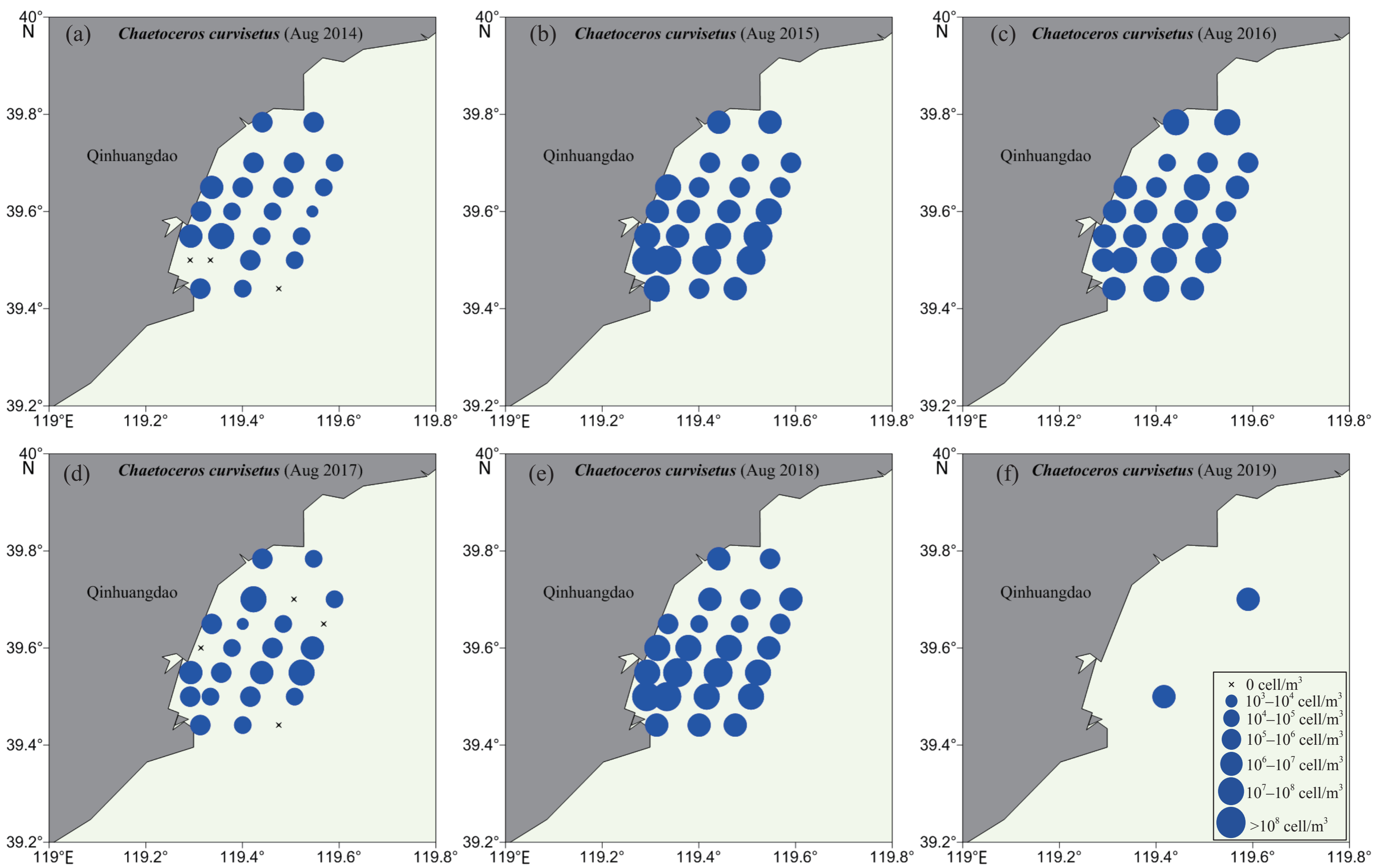

Supplement: Supplementary file 1 [file life-13-00192-s001.zip › Figure S2.pdf]

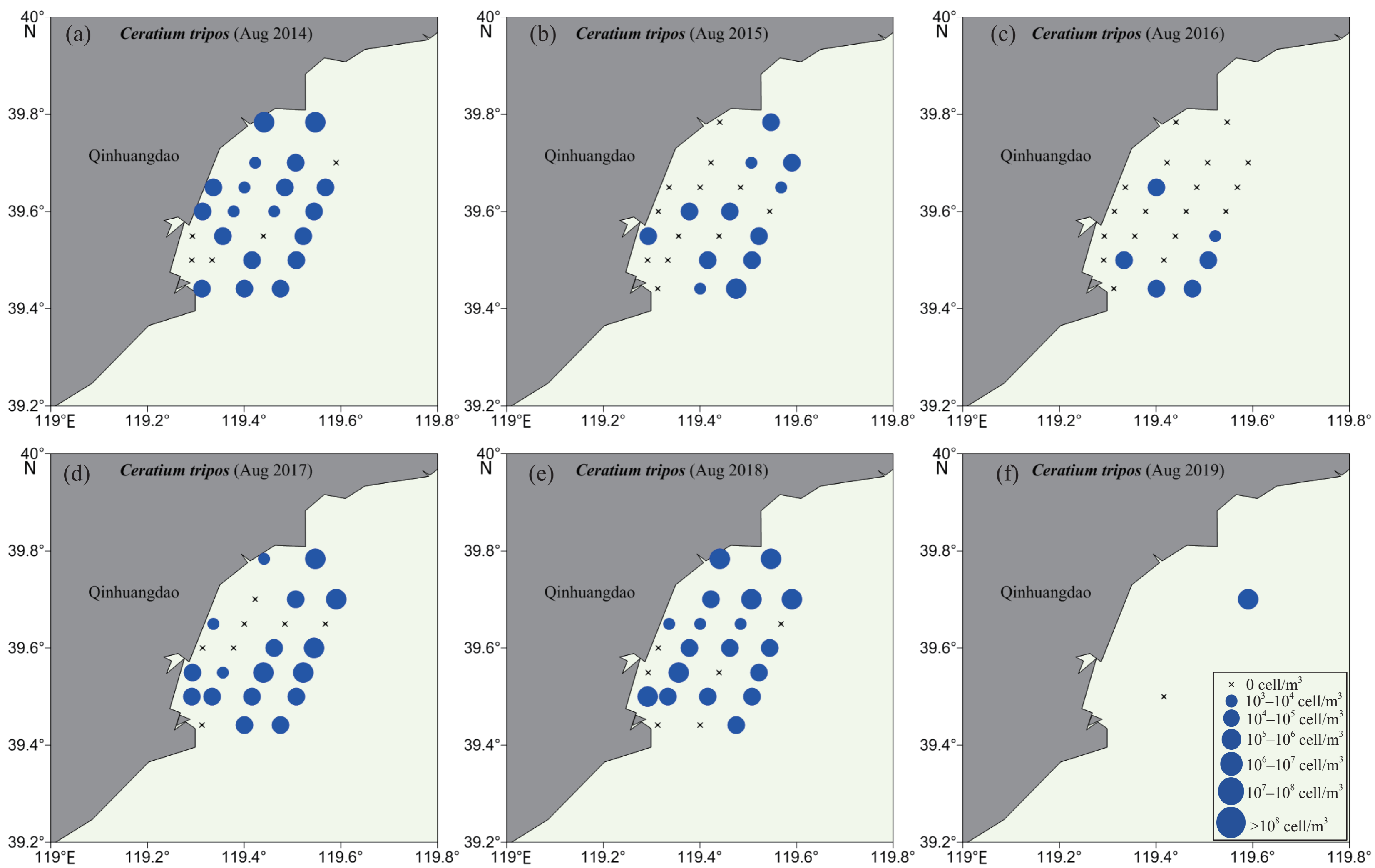

Supplement: Supplementary file 1 [file life-13-00192-s001.zip › Figure S3.pdf]

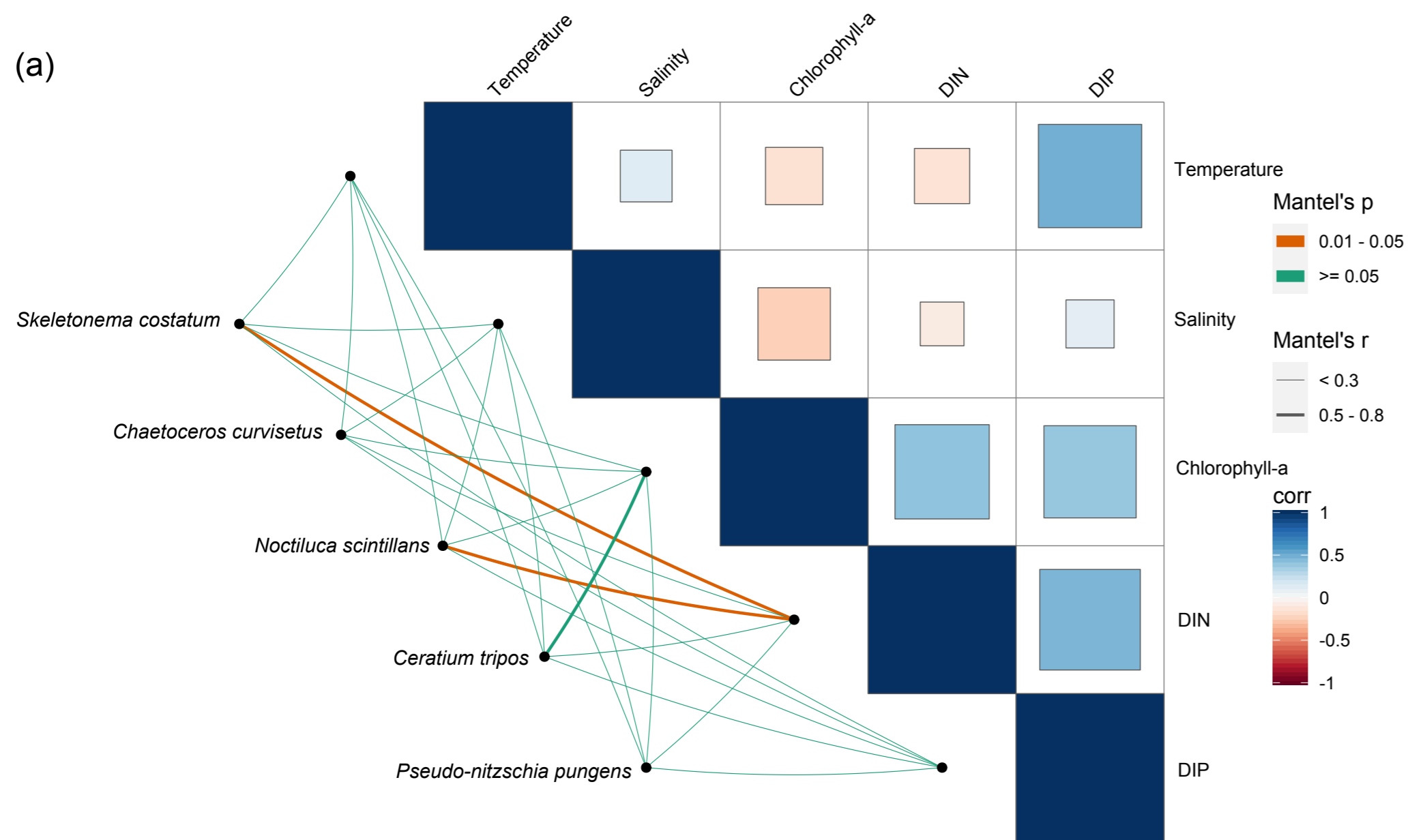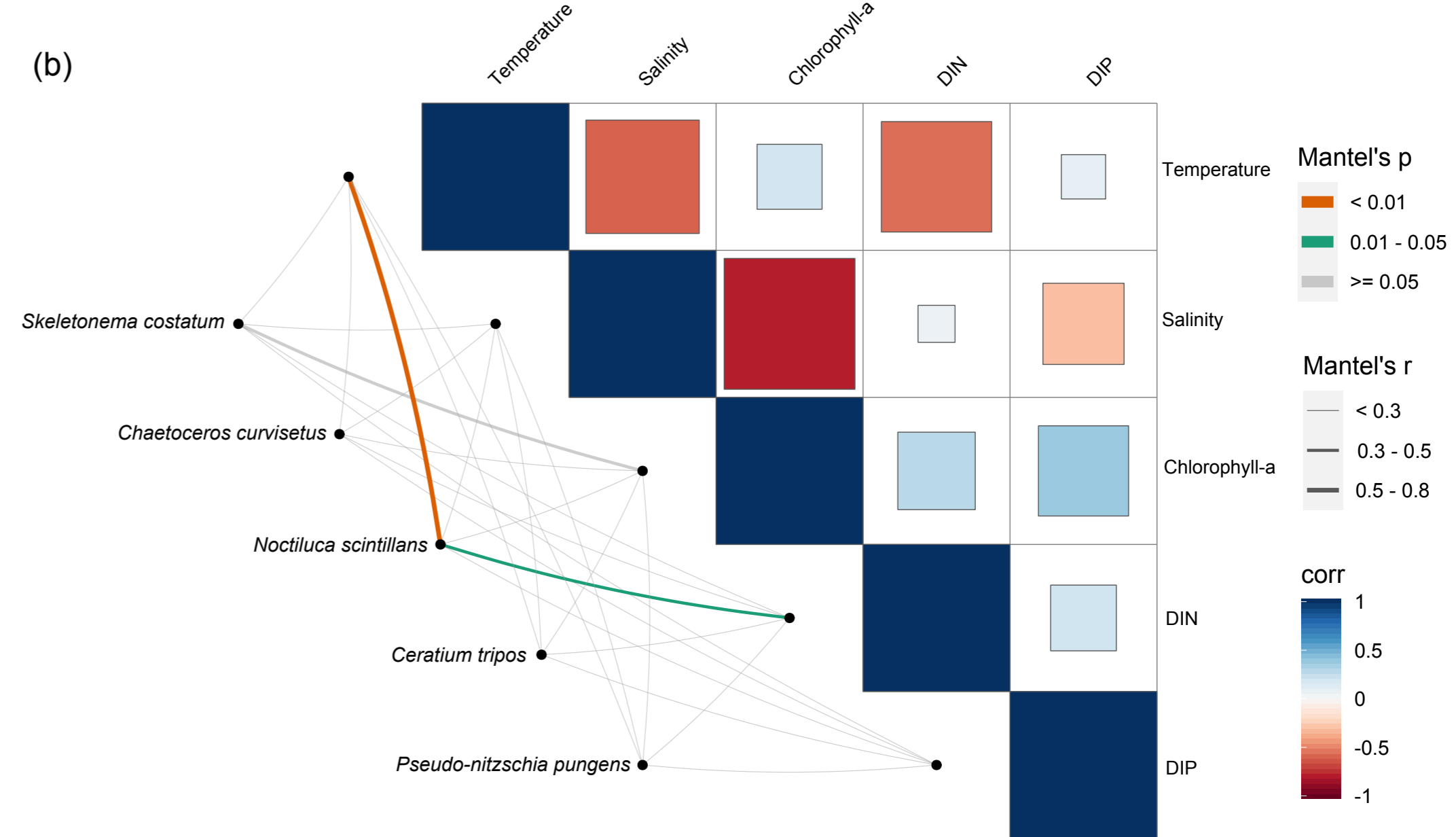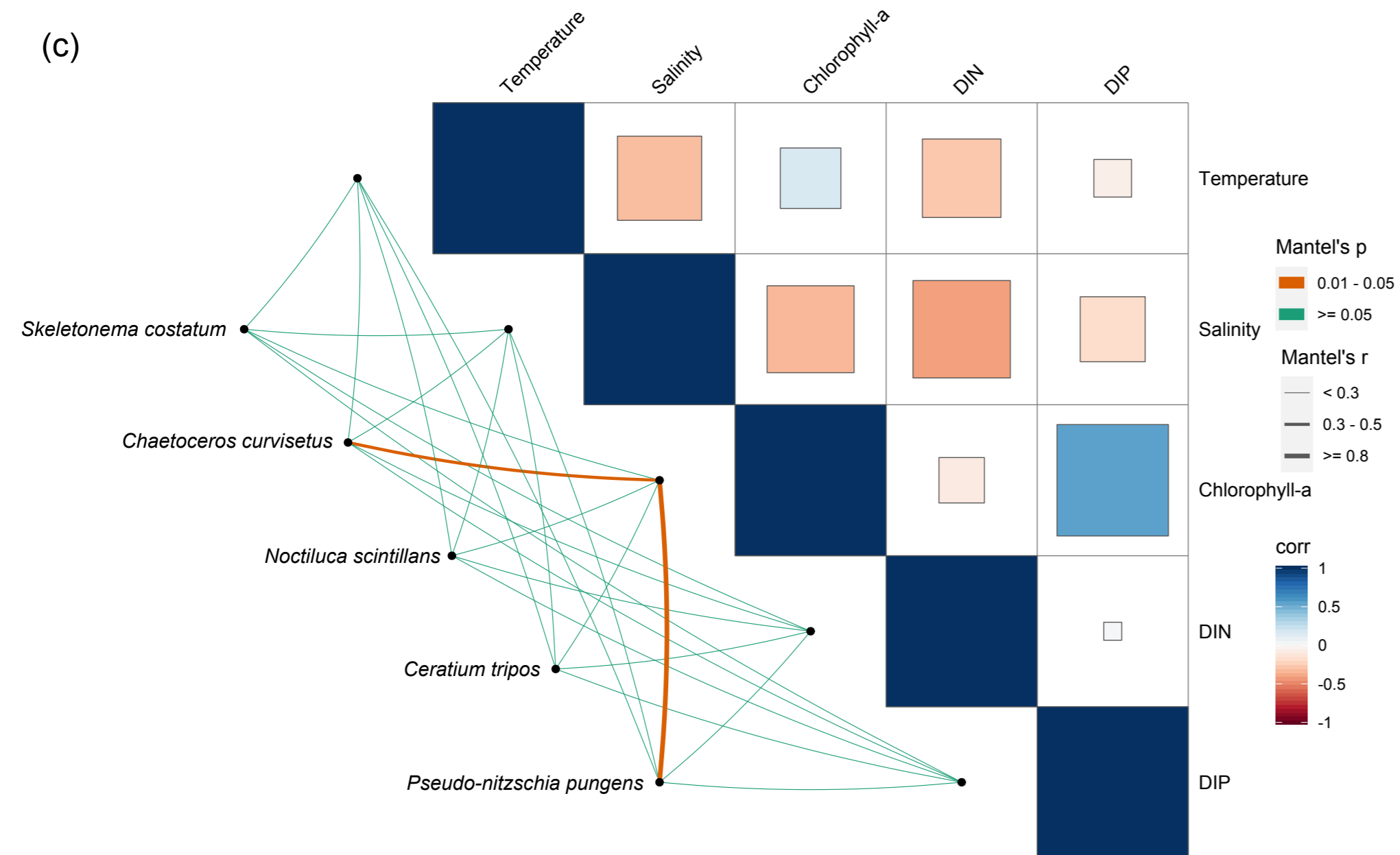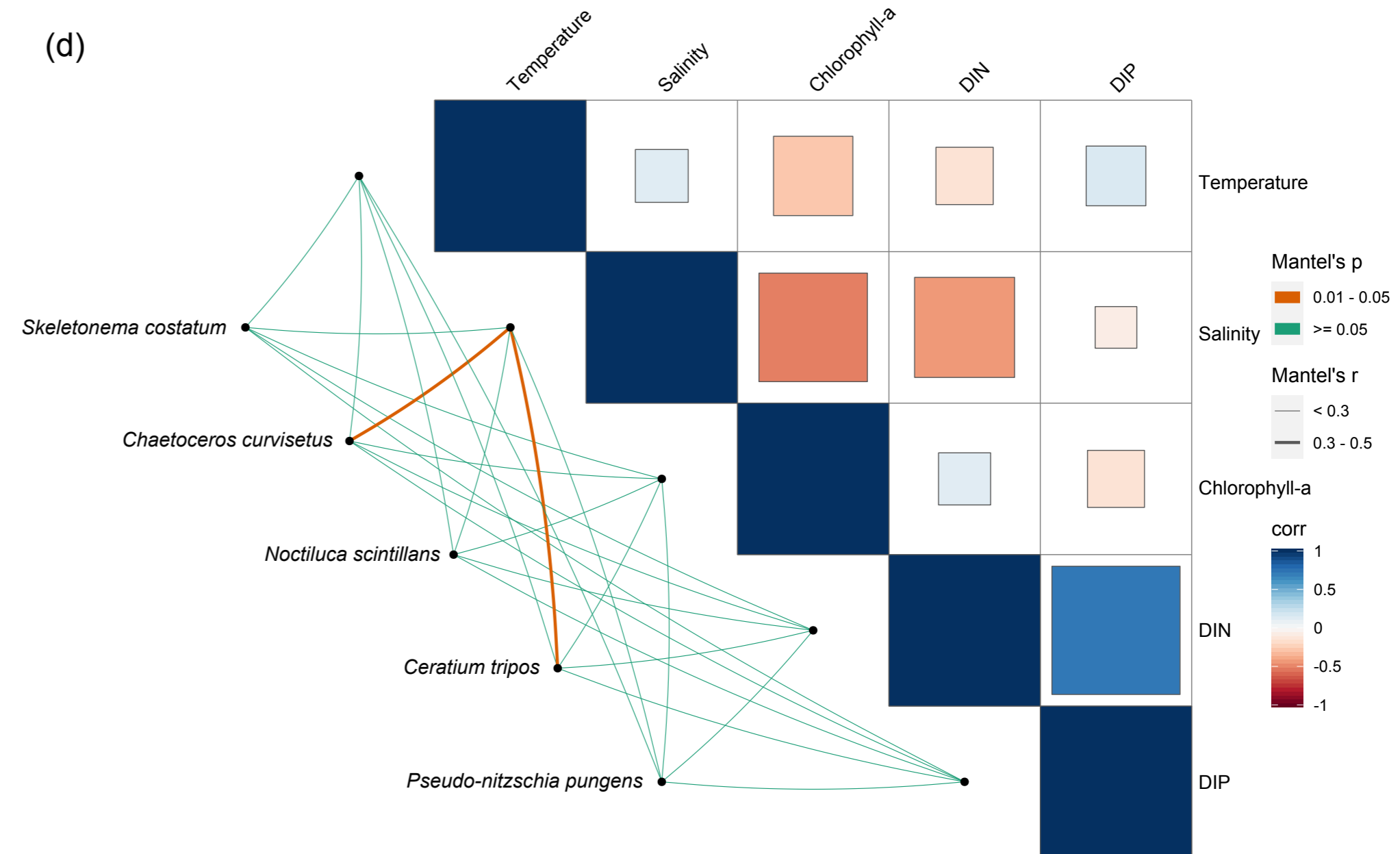

Supplement: Supplementary file 1 [file life-13-00192-s001.zip › Figure S4.pdf]
